# Olig2 regulates Purkinje cell generation in the early developing mouse cerebellum

**DOI:** 10.1038/srep30711

**Published:** 2016-07-29

**Authors:** Jun Ju, Qian Liu, Yang Zhang, Yuanxiu Liu, Mei Jiang, Liguo Zhang, Xuelian He, Chenchen Peng, Tao Zheng, Q. Richard Lu, Hedong Li

**Affiliations:** 1 West China Developmental & Stem Cell Institute, Department of Obstetric & Gynecologic and Pediatric, Key Laboratory of Obstetric & Gynecologic and Pediatric Diseases and Birth Defects, Ministry of Education, West China Second University Hospital, Sichuan UniversityChengdu, 610041, P.R. China; 2 School of Life Science, Sichuan University, Chengdu 610041, P.R. China; 3 Department of Pediatrics, Cincinnati Children’s Hospital Medical Center, Cincinnati, Ohio 45229, USA

## Abstract

The oligodendrocyte transcription factor *Olig2* plays a crucial role in the neurogenesis of both spinal cord and brain. In the cerebellum, deletion of both *Olig2* and *Olig1* results in impaired genesis of Purkinje cells (PCs) and Pax2^+^ interneurons. Here, we perform an independent study to show that Olig2 protein is transiently expressed in the cerebellar ventricular zone (VZ) during a period when PCs are specified. Further analyses demonstrate that Olig2 is expressed in both cerebellar VZ progenitors and early-born neurons. In addition, unlike in the ganglionic eminence of the embryonic forebrain where Olig2 is mostly expressed in proliferating progenitors, Olig2^+^ cells in the cerebellar VZ are in the process of leaving the cell cycle and differentiating into postmitotic neurons. Functionally, deletion of *Olig2* alone results in a preferential reduction of PCs in the cerebellum, which is likely mediated by decreased neuronal generation from their cerebellar VZ progenitors. Furthermore, our long-term lineage tracing experiments show that cerebellar *Olig* gene-expressing progenitors produce PCs but rarely Pax2^+^ interneurons in the developing cerebellum, which opposes the “temporal identity transition” model of the cerebellar VZ progenitors stating that majority of Pax2^+^ interneuron progenitors are transitioned from Olig2^+^ PC progenitors.

The basic helix-loop-helix (bHLH) transcription factors *Olig* genes, i.e. *Olig1* and *Olig2*, are first identified in the spinal cord and named as such because of their pivotal function in oligodendrocyte development throughout the central nervous system (CNS)^1^,^2^. Subsequently, the two groups who discovered the genes reported simultaneously that *Olig2* and *Olig1* are also required for spinal motor neuron (MN) specification^3^,^4^. Similarly in the developing forebrain, *Olig2*-expressing progenitors in the medial ganglionic eminence (MGE) give rise to subpopulations of GABAergic interneurons where the homeobox transcription factor Dlx2 counteracts with Olig2 to specify neuronal versus oligodendroglial cell fate^5^. In addition, at least some cholinergic neurons in the basal forebrain are also derived from *Olig2*-expressing progenitors, and *Olig2* deletion leads to a severe reduction of these neurons^6^.

In the cerebellum, Purkinje cells (PCs) are GABAergic projection neurons that, along with deep cerebellar nuclei (DCN) GABAergic projection neurons, are derived from the ventricular zone (VZ) of the early cerebellar primordium^7^. GABAergic inhibitory interneurons are also derived from the VZ, and yet in a more ventral region that can be defined by Pax2 expression^8^. Importantly, pancreas transcription factor 1a (Ptf1a) plays an indispensable role in the generation of all VZ-derived cerebellar GABAergic neurons including PCs and Pax2^+^ interneurons^9^,^10^. Although several other transcription factors, namely Mash1^11^, Ngn1^12^, Ngn2^13^, NeuroD1^14^, Gsx1^15^ and Olig1, 2^14^,^15^ have been described to express in the cerebellar VZ with distinct micro-domains^16^, the mechanisms involved in the specification and generation of VZ-derived GABAergic neurons including PCs are still partially understood. In a recent report, Seto *et al*. demonstrated that Olig2 counteracts with Gsx1 in the early cerebellar VZ to maintain PC progenitor identity, and that deletion of both *Olig1* and *Olig2* leads to reduction of PCs and increase of Pax2^+^ interneurons while deletion of *Olig2* alone shows no obvious phenotypic defects^15^. However, in the present study, we show that deletion of *Olig2* alone results in a significant reduction of PCs and no change of Pax2^+^ interneurons, indicating that Olig2 function is required for a complete specification of PCs. Mechanistically, we also show that Olig2 is expressed in the late-phase of the VZ progenitor cell cycle and controls the rate of neurogenesis from cerebellar VZ progenitors, but not their proliferation. Furthermore, our long-term lineage tracing analysis indicates that Olig2^+^ progenitors give rise to PCs and DCN neurons, but rarely Pax2^+^ interneurons, challenging the “temporal identity transition” model of the cerebellar VZ progenitors that was recently proposed^15^.

## Results

### Olig2 is co-expressed with neuronal and progenitor markers in the early cerebellum

**Figure 1:**
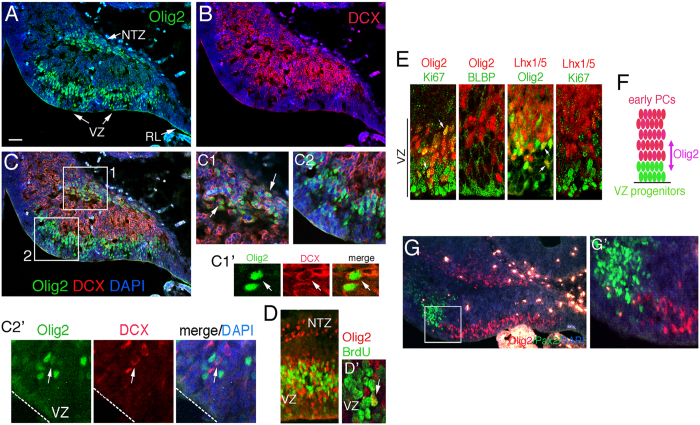
Differential neuronal expression patterns of Olig2^+^ cells in the early embryonic cerebellum. Co-immunostaining is performed to analyze the expression of Olig2 (**A**) and a neuronal marker, DCX (**B**) on sagittal sections of the E12.5 cerebellum. The overlay image (**C**) reveals differential DCX expression patterns of the Olig2^+^ cells in the VZ and NTZ. The enlarged images of the boxed regions in (**C**) are shown in (**C1**) and (**C2**), respectively. Double-positive cells are pointed by arrows in (**C1**) and an arrow in a higher power confocal image (**C1’**). Double-positive cells (indicated by an arrow in **C2’**, a higher power confocal image of **C2** region) are very rarely seen in (**C2**) where Olig2 and DCX show mostly a non-overlapping staining pattern. (**D**) Co-immunostaining of Olig2 and BrdU in the VZ of the E12.5 cerebellum that has been treated by a BrdU pulse-labeling. (**D’**) The confocal image of the VZ in (**D**) showing a double-positive cell (arrow). (**E**) Co-expression analysis of Olig2 with markers of proliferation (Ki67), radial glia (BLBP) and PC differentiation (Lhx1/5) in the cerebellar VZ at E13.5. (**F**) A diagram depicting Olig2 expression range during the transition of VZ progenitors to early PCs. (**G**) Co-immunostaining with anti-Olig2 and anti-Pax2 on sagittal cerebellar sections of the E13.5 wild-type mouse. (**G’**) is the enlarged image of boxed regions in (**G**) showing a non-overlapping staining pattern. VZ, ventricular zone; RL, rhombic lip; NTZ, nuclear transitory zone. Scale bar: 80 μm in **A**,**B**,**C** and **G**; 25 μm in **C1** and **C2**; 15 μm in **C1’** and **D’**; 20 μm in **C2’**, **E** and **G’**; 40 μm in **D**.

Olig2 is transiently expressed in the cerebellar VZ during E11.5∼E13.5, a time window of PC specification^14^. To further delineate neurogenic function of Olig2 in the early cerebellum, we performed a co-staining of Olig2 with a marker of early postmitotic neurons, Doublecortin (DCX), at E12.5 when Olig2 expression is strong (Fig. 1A). We found that DCX staining is prevalent in the cerebellar plate at this stage but absent from the rhombic lip (RL) and the VZ, the two major germinal zones of the developing cerebellum (Fig. 1B). The nuclear transitory zone (NTZ) Olig2^+^ cells mostly co-express DCX suggesting that they are postmitotic neurons (Fig. 1C,C1). In contrast, Olig2 expression in the VZ shows a largely non-overlapping pattern with DCX (Fig. 1C2). Occasionally, we were able to find DCX and Olig2 double-positive cells at the boundary between the DCX^+^ and Olig2^+^ zones (Fig. 1C2’, arrow), suggesting that DCX^+^ neurons are derived from VZ Olig2^+^ progenitors by downregulating Olig2 expression. Similar expression pattern has been found with Olig2 and an early postmitotic neuronal marker Lhx1/5^17^,^18^ previously^14^. A 2-hour bromodeoxyuridine (5-bromo-2′-deoxyuridine, BrdU)-pulse labeling analysis showed that 25.2 ± 3.3% of the VZ Olig2^+^ cells are also BrdU^+^ (Fig. 1D and arrow in 1D’) confirming that they are dividing progenitors, whereas the NTZ Olig2^+^ cells are nearly all BrdU^−^ (Fig. 1D). Therefore, the dynamic expression pattern of Olig2 during early cerebellar development suggests its potential role in the genesis and differentiation of several cerebellar neuronal types including PCs that are differentiated from the VZ progenitors at this developmental stage.

Next, we examined Olig2 expression in the VZ in further detail to determine its potential function in PC generation. In the E13.5 cerebellar VZ, a small fraction (7.1 ± 3.6%) of Olig2^+^ cells co-express the proliferation marker Ki67 (Fig. 1E). In addition, we found that the Olig2^+^/Ki67^+^ cells are rarely epithelial progenitors that can be labeled by Ki67 and a radial glial marker brain lipid binding protein (BLBP) (Fig. 1E). On the other hand, 35.9 ± 3.6% of Olig2^+^ cells are positive for an early marker of postmitotic PCs, Lhx1/5, which shows a non-overlapping expression pattern with Ki67 (Fig. 1E). Therefore, these immunostaining data indicate that Olig2 is expressed in both non-epithelial VZ progenitors and newborn PCs (Fig. 1F), defining the specification process of PCs from the VZ progenitors. Similar expression patterns have also been found with some of these markers in the E12.5 cerebellum previously^14^,^15^.

Besides PCs, GABAergic inhibitory interneurons are also generated from cerebellar VZ and can be identified by their expression of Pax2^8^. We found that Pax2 and Olig2 exhibit a nearly non-overlapping expression pattern at E13.5 (Fig. 1G,G’) similarly to E12.5^15^ suggesting that Olig2 plays little role in the specification of GABAergic inhibitory interneurons.

### The NTZ Olig2^+^ cells represent a distinct subpopulation of DCN neurons in the developing cerebellum

The expression pattern of Olig2 in the NTZ of the developing cerebellum suggests that these positive cells are derived from the RL. In addition, our result indicates that these cells are DCX^+^ newborn neurons (Fig. 1). To further characterize the NTZ Olig2^+^ cells, we performed co-immunostaining analyses with anti-Olig2 and markers of RL derivatives. A previous report has indicated that Pax6, Tbr2 and Tbr1 are expressed in differentiating gltutamatergic DCN neurons in a sequential manner^19^. At E12.5 when Olig2 is highly expressed in the NTZ, we found that some Pax6^+^ cells are sparsely distributed along the dorsal pial surface, and that Pax6^+^ and Olig2^+^ cells do not overlap (data not shown). As development proceeds, Pax6^+^ cells are more prominent along the pial surface as shown at E14.5 (Fig. S1A). However, the NTZ Olig2 and Pax6 still show a non-overlapping pattern (Fig. S1A, insert). Between the Olig2^+^ and Pax6^+^ domains, there apprear to be a group of cells that are negative for both markers, but positive for Tbr1 (Fig. S1B). Although the Olig2^+^ and Tbr1^+^ domains are closer in position with double positive cells being occasionally seen (Fig. S1B, insert, arrowhead), they are also largely non-overlapping. In addition, the NTZ Olig2^+^ cells are nearly all postmitotic as indicated in a 2-hour BrdU-pulse labeling experiment at E14.5 (Fig. S1C). Notably, a significant number of BrdU^+^ cells can still be found in the VZ, and yet Olig2 expression has already disappeared in this region at this developmental stage (Fig. S1C). At E18.5, while Tbr1^+^ neurons concentrate to the medial part of the DCN, Olig2 expression is more towards the lateral region (Fig. S1D). Two morphologically distinct Olig2^+^ cells are found with smaller cells being located in the periphery of the Olig2^+^ domain and bigger cells being in the core (Fig. S1D, inserts). While the small Olig2^+^ cells are often NG2^+^ suggestive of oligodendrocyte progenitor cells (OPCs), the big Olig2^+^ cells are NeuN^+^ (data not shown). In sum, these expression patterns suggest that Olig2 expression labels a distinct group of DCN neurons that locate more lateral to the Tbr1^+^ neurons in the DCN, which is consistent with a previous observation^14^.

### *Olig2*-null mice show reduced PC generation

**Figure 2:**
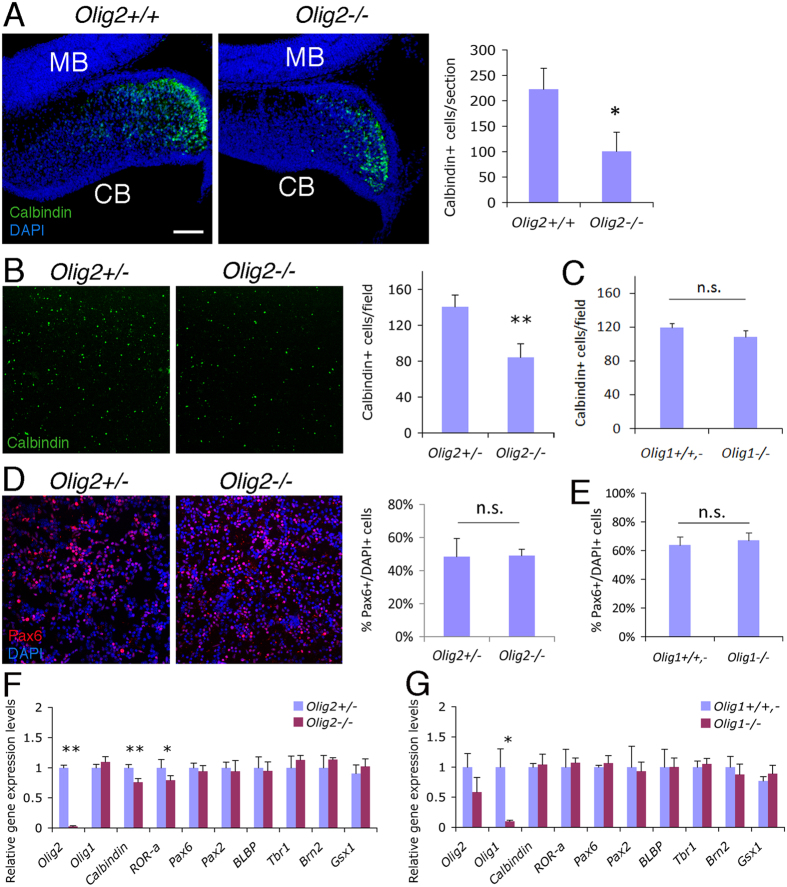
*Olig2*-null mice show reduced PC generation. (**A**) Immunostaining with an anti-Calbindin antibody on E15.5 cerebellar mid-sagittal sections of *Olig2*+/+ and *Olig2*−/− mice shows notably reduction of PCs in the cerebellum (CB). Quantification on confocal images shows a significant reduction in the number of Calbindin^+^ cells per cerebellar section in *Olig2*−/− mice compared with controls (n = 3 for both genotypes). DAPI is used to label nuclei. MB, midbrain. The number of Calbindin^+^ cells per field in the acute cultures of dissociated E17.5 *Olig2*−/− (**B**) and E18.5 *Olig1*−/− (**C**) cerebella are compared with their controls (n = 3 for both mutant and control genotypes). In these same acute cultures, the percentage of Pax6^+^ cells among total cells (DAPI^+^) are also compared for E17.5 *Olig2*−/− (**D**) and E18.5 *Olig1*−/− (**E**) cerebella. Gene expression levels in dissected cerebellar tissues are compared by qRT-PCR between *Olig2*+/− and *Olig2*−/− mice (n = 4 for both genotypes) at E18.5 (**F**), or between *Olig1*+/+,− and *Olig1*−/− mice (n = 3 for both genotypes) at E17.5 (**G**). Scale bar: 200 μm in **A** and **B**; 100 μm in **D**. *P < 0.05; **P < 0.01; n.s., not significant by Student’s *t*-test.

**Figure 3:**
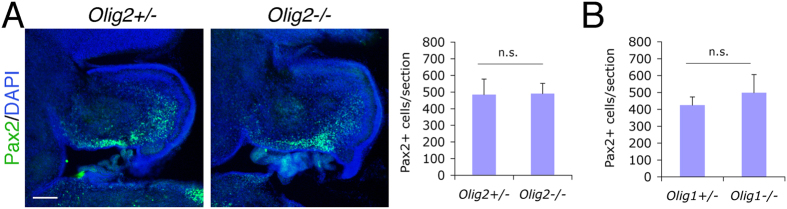
No significant change in Pax2^+^ interneuron progenitors in the *Olig2*-null cerebellum. (**A**) Immunnostaining analysis is performed on mid-sagittal cerebellar sections of the E18.5 *Olig2*-null mice. The number of Pax2^+^ cells per cerebellar section is quantified and compared. No significant change in the number of Pax2^+^ interneuron progenitors is detected between *Olig2*-null and control mice (n = 3 for both genotypes). (**B**) Similar immunostaining was also performed and quantified on cerebellar sections of E18.5 *Olig1*-null mice. DAPI is used to label nuclei. Scale bar: 200 μm. n.s., not significant by Student’s *t*-test.

To explore directly the function of Olig2 during cerebellar neurogenesis, we examined mice carrying an *Olig2*-null mutation. Since *Olig2*-null mice die soon after birth^3^, neurogenesis has to be analyzed during embryonic stages in this animal. First, immunostaining with an antibody against Calbindin, a specific PC marker, showed a siginificant reduction in PC number on mid-sagittal sections of *Olig2*-null cerebella at E15.5 compared with controls (Fig. 2A). Normally, PCs that are generated from ventral cerebellar VZ migrate dorsally and spread tangentially underneath the cerebellar pial surface around this developmental stage^20^. We observed numerous Calbindin^+^ cells within the cerebellar parenchyma in the control mouse (presumably the migrating PCs), but very few in the *Olig2*-null mouse (Fig. 2A). This observation may suggest that PC migration is disrupted in the absence of Olig2. However, this could also be explained by reduced number of PCs and thus early termination of the migration process in the *Olig2*-null cerebella. Second, to confirm that the PC reduction on mid-sagittal cerebellar sections is not due to a redistribution of PC in the *Olig2*-null cerebellum, we examined acute cultures that were dissociated from whole E17.5 cerebellar tissues and demonstrated a significant decrease in the number of Calbindin^+^ cells per field in *Olig2*-null cultures (Fig. 2B), but not in *Olig1*-null cultures (Fig. 2C), while the percentage of Pax6^+^ granule neuron precursors among total cells showed no change in either mutant cultures compared with controls (Fig. 2D,E). These results indicate a specific effect of *Olig2* ablation on PC generation. Lastly, gene expression comparison at E18.5 indicated that the mRNA levels of *Calbindin* and another PC marker *RORα*^21^ are significantly reduced in *Olig2*-null cerebella, while *Pax2* and *Pax6* mRNA levels remain unchanged (Fig. 2F). Furthermore, the levels of the Bergmann glial marker *BLBP* and the DCN neuron markers *Tbr1* and *Brn2*^19^ remained unchanged in the *Olig2*-null cerebella indicating that *Olig2* ablation has little effect on these two cell types (Fig. 2F). *Gsx1* has been shown to promote Pax2^+^ interneuron generation^15^ and did not display a change in expression (Fig. 2F). The effect of *Olig1* ablation on cell type marker expression was also analyzed at E17.5, and, except for *Olig1* itself, no significantly changed genes were observed between *Olig1*-null and control cerebellar samples (Fig. 2G). To further confirm the unchanged level of *Pax2* expression in *Olig2*-null cerebella (Fig. 2F), we also performed immunostaining on E18.5 mid-sagittal cerebellar sections and found no significant difference in the number of Pax2^+^ cells per section between *Olig2*-null and control cerebella (Fig. 3A). Pax2^+^ cells were also quantified on E18.5 cerebellar sections of the *Olig1*-null mice and showed no significant difference compared with controls (Fig. 3B). Taken together, *Olig2*, but not *Olig1*, ablation specifically reduces PC generation and has no detectable effects on other neuronal types and astroglia in the embryonic cerebellum.

### Olig2 deletion impairs PC generation from their cerebellar VZ progenitors without affecting overall cell proliferation

**Figure 4:**
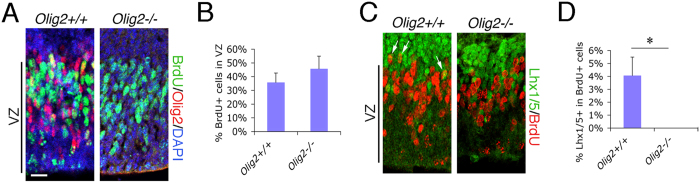
*Olig2*-deletion induces impaired PC differentiation from cerebellar VZ progenitors without affecting proliferation. (**A**) Proliferation of cerebellar VZ progenitors at E12.5 is analyzed by their ability to incorporate BrdU in a 2-hour pulse-labeling treatment and compared between *Olig2*+/+ and *Olig2*−/− mice. (**B**) The percentage of BrdU^+^ cells among total cells (DAPI^+^) in the cerebellar VZ (as indicated) is calculated and compared between the two genotypes (n = 3 for both genotypes). (**C**) PC generation from the proliferating cerebellar VZ progenitors at E12.5 is also analyzed by co-staining of the postmitotic neuronal marker Lhx1/5 and BrdU in *Olig2*+/+ and *Olig2*−/− mice. (**D**) Percentage of Lhx1/5^+^ cells among BrdU^+^ cells in the dorsal portion of the cerebellar VZ is quantified and compared between the two genotypes (n = 3 for both genotypes). VZ, ventricular zone. Scale bar: 20 μm in **A** and **C**. *P<0.05 by Student’s *t*-test.

To check if *Olig2*-ablation affects progenitor proliferation in the cerebellar VZ, we performed a 2-hour BrdU-pulse labeling at E12.5 and compared BrdU-labeled cells. We found no significant difference in the percentage of BrdU^+^ cells among total cells between *Olig2*-null and wild-type animals (Fig. 4A,B). The co-staining of BrdU and the postmitotic neuronal marker Lhx1/5 in the dorsal portion of cerebellar VZ at this stage allowed us to identify newborn PCs during the 2-hour BrdU labeling period. In wild-type mice, we observed a small number of BrdU and Lhx1/5 double-positive cells that are usually localized along the boundary between BrdU and Lhx1/5-positive zones in the cerebellar VZ (Fig. 4C, arrows). However, we could hardly detect any such double-positive cells in *Olig2*-null mice, despite that numerous Lhx1/5^+^ PCs are present (Fig. 4C,D). In addition, we detected very few caspase-3^+^ cells in the E12.5 cerebella of both types of mice (data not shown) suggesting that apoptosis is not involved in the PC defect of *Olig2*-null mice. Thus, Olig2 may play little role in progenitor proliferation *per se*, but is required for normal rate of PC generation from their progenitors in the cerebellar VZ. In a given time period of PC generation, fewer PCs may be specified from VZ progenitors and therefore moderate loss of PCs in the *Olig2*-null cerebellum (Fig. 2).

### Unlike in the embryonic forebrain, Olig2-expressing progenitors in the cerebellar VZ are in the late phase of the cell cycle

**Figure 5:**
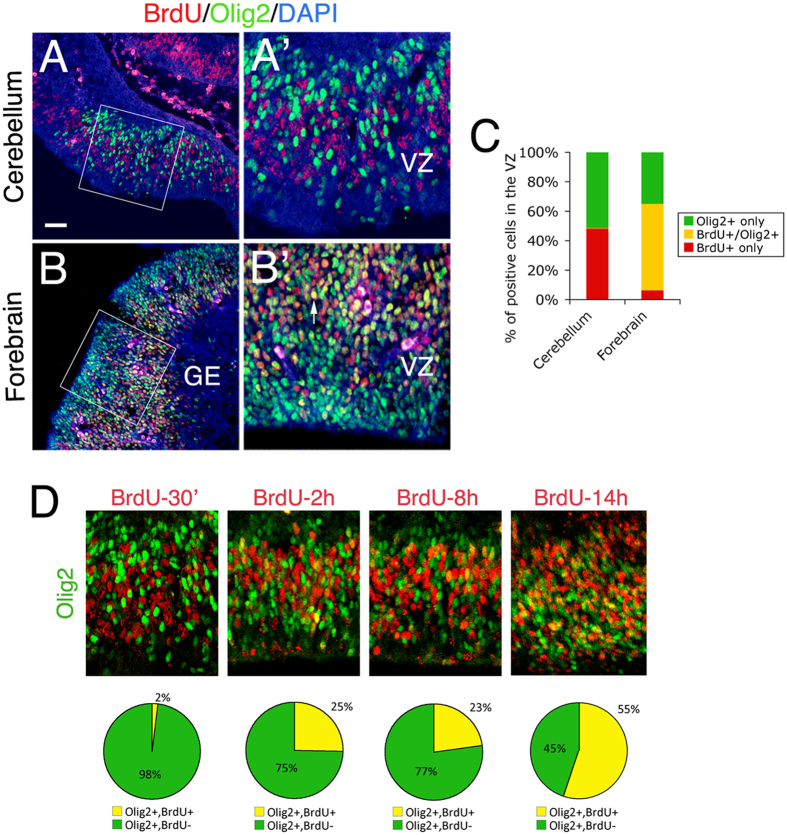
Differential expression patterns of Olig2 in the VZ progenitors relative to cell cycle in the E12.5 cerebellum and forebrain. Co-immunostainings were done on mid-sagittal sections of the cerebellum (**A**) and forebrain (**B**) of E12.5 embryos that have been pulse-labeled with BrdU for 30 min before sacrifice. (**A’,B’**) are enlarged images of boxed regions in (**A**,**B**), respectively. The arrow indicates a double-positive cell in (**B’**). (**C**) Comparison of BrdU labeling in the VZ Olig2^+^ cells between cerebellum and forebrain. Note that BrdU labels more than 50% of the Olig2^+^ cells (BrdU^+^/Olig2^+^) in the GE of the forebrain, but nearly none in the cerebellar VZ. (**D**) A series of BrdU-pulse labeling analyses of E12.5 cerebellar VZ progenitors. Percentages are shown to represent BrdU^+^ and BrdU^-^ fractions among Olig2^+^ cells in the cerebellar VZ. VZ, ventricular zone; GE, ganglionic eminence.

To further examine the proliferative characteristic of the Olig2-expressing progenitors in the cerebellar VZ, we performed a 30-min BrdU-pulse labeling at E12.5 to label exclusively the cells in the S-phase of the cell cycle. Consistent with our observation by immunostaining that Olig2 is expressed in progenitors that are leaving the cell cycle (Fig. 1), we found that Olig2-expressing cells are rarely labeled by BrdU in the 30-min pulse (Fig. 5A,A’). On the contrary, nearly 60% of the Olig2^+^ cells are labeled by BrdU in the ganglionic eminence (GE) of the forebrain of the same animal (Fig. 5B,B’), suggesting that most Olig2^+^ cells in the forebrain are progenitors in the S-phase, actively dividing. Furthermore, a more detailed cell cycle analysis of E12.5 cerebellar VZ progenitors by BrdU-pulse labeling shows that Olig2 is preferentially expressed in the late phase of the progenitor cell cycle (14h after BrdU pulse) (Fig. 5D). Therefore, these results confirm that Olig2-expressing progenitors in the cerebellar VZ are distinct from the ones in other CNS regions and may exhibit unique neurogenic properties during development.

### *Olig* gene-expressing progenitors give rise to PCs and DCN neurons, but rarely Pax2^+^ interneurons, in the cerebellum

**Figure 6:**
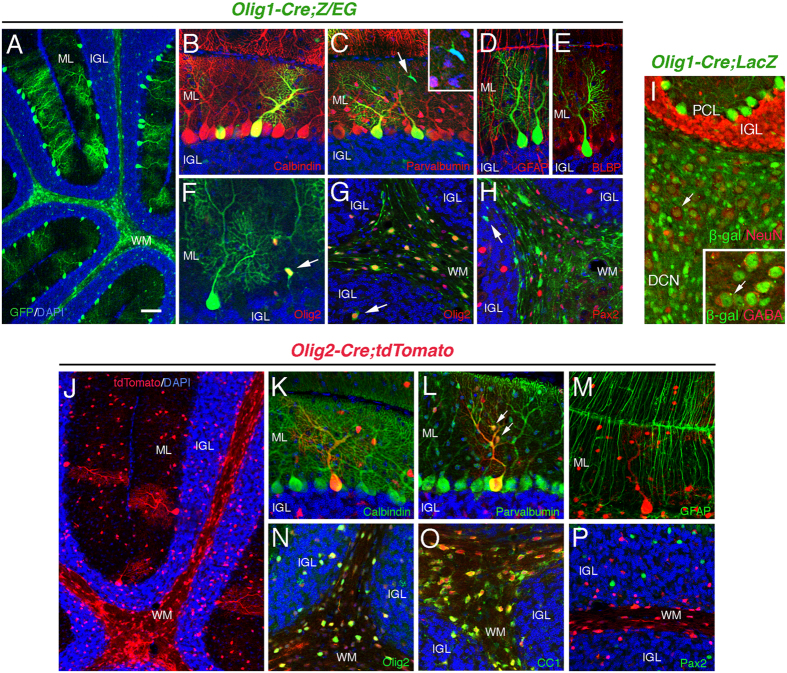
*Olig* gene-expressing progenitors give rise to PCs and DCN neurons but rarely Pax2^+^ interneurons in the postnatal cerebellum as revealed by long-term lineage tracing. *Olig1-Cre* lineage tracing analysis is performed in the cerebellum of either *Z/EG* (**A–H**) reporter line at P18 or *ROSA26;LacZ* (**I**) reporter line at P16. Labeled cells with different morphology in the cerebellum of *Olig1-Cre;Z/EG* mice are revealed by their GFP expression (**A**). The identity of GFP^+^ cells are examined by co-staining with cell-type specific markers Calbindin (**B**), Parvalbumin (**C**), GFAP (**D**), BLBP (**E**), Olig2 (**F,G**) and Pax2 (**H**). Some GFP^+^ cells are indicated as either negative (arrows in **C** and **H**) or positive (arrows in **F,G**) for marker staining. The high magnification insert in (**C**) confirms the cellular identity of the indicated GFP^+^ cell by showing the presence of DAPI^+^ nucleus. Many labeled cells in the cerebellar DCN of *Olig1-Cre;LacZ* reporter mice are observed by anti-β-gal staining, some of which are NeuN^+^ (arrow, **I**) and GABA^+^ (arrow, insert in **I**). *Olig2-Cre* lineage tracing analysis is also performed in the cerebellum of *ROSA26;tdTomato* (**J–P**) reporter line at P22. Labeled cells with different morphology in the cerebellum of *Olig2-Cre;tdTomato* mice are revealed by their tdTomato expression (**J**). The identity of tdTomato^+^ cells are examined by co-staining with cell-type specific markers Calbindin (**K**), Parvalbumin (**L**), GFAP (**M**), Olig2 (**N**), CC1 (**O**) and Pax2 (**P**). Note that two tdTomato^+^ cells are co-labeled with Parvalbumin suggestive of small interneurons (arrows in **L**). DAPI is used to label nuclei. ML, molecular layer; IGL, internal granule layer; WM, white matter; DCN, deep cerebellar nuclei. Scale bar: 100 μm in (**A,J**); 20 μm in (**B–H**,**K–P**); 25 μm in **I**.

To obtain a full spectrum of neuronal types that may involve Olig2 function in the developing cerebellum, we performed a long-term lineage tracing analysis using both *Olig1-Cre* and *Olig2-Cre* reporter mouse lines. Since *Olig1* and *Olig2* expressions are coordinately regulated in the developing CNS^22^ and are co-expressed in the cerebellum^15^, we expected to see similar labeling patterns in these reporter lines. The *Olig1-Cre* faithfully labels oligodendrocyte lineage cells throughout the CNS in our previous studies^23^,^24^. Indeed, in the P18 cerebellum of the *Olig1-Cre;Z/EG* reporter mouse, we observed many GFP^+^ cells in the white matter (WM) where most oligodendrocytes reside (Fig. 6A). Besides, we also observed strong GFP^+^ cells with big cell bodies and complex dendritic arbors in the molecular layer (ML) (Fig. 6A). Further immunohistochemical analysis confirmed their identity as PCs that are both Calbindin^+^ and Parvalbumin^+^ (Fig. 6B,C), but negative for Bergmann glial markers, GFAP (Fig. 6D) and BLBP (Fig. 6E). The rarely seen small GFP^+^ cells in the ML are Parvalbumin^−^ (Fig. 6C, arrow and insert), but Olig2^+^ (Fig. 6F, arrow), indicative of cells of oligodendrocyte lineage, but not small interneurons. Nearly all GFP^+^ cells in the WM co-express Olig2 (Fig. 6G) but none co-express Pax2, a pan marker of GABAergic inhibitory interneurons (Fig. 6H). Next, we examined if *Olig1-Cre* could label any neuronal cell types in the DCN. For that purpose, we engaged the *Olig1-Cre;LacZ* reporter line where cellular labeling revealed by anti-β-gal staining is mostly nuclear and easy to identify. In the DCN of this reporter line, a significant number of big β-gal^+^ cells are NeuN^+^ (Fig. 6I, arrow), some of which are also GABA^+^ (Fig. 6I, insert, arrow) suggestive of GABAergic DCN projection neurons. One thing to note is that we do observe variable labeling efficiency between the two reporter lines. For example, the labeling efficiency of PC is higher in the *Olig1-Cre;LacZ* (65.5 ± 3.8%) than in the *Olig1-Cre;Z/EG* reporter line (31.6 ± 7.6%). We reason that this variation may be due to the distinct genetic structure of the reporter loci in each reporter mouse line. Nevertheless, these results indicate that most, if not all, PCs are derived from *Olig* gene-expressing progenitors in the cerebellum. In parallel, we also performed lineage tracing analysis with the *Olig2-Cre;tdTomato* reporter line, in which labeled cerebellar cell types can be identified by their expression of tdTomato (Fig. 6J). Again, PCs are identified as a labeled cell type by their expression of Calbindin and Parvalbumin (Fig. 6K,L) but not GFAP (Fig. 6M). Interestingly, some small tdTomato^+^ cells in the ML of this reporter line shows co-expression of Parvalbumin (arrows, Fig. 6L) suggestive of small interneuron identity. In the WM, *Olig2-Cre*-labeled cell types are nearly all of oligodendrocyte lineage being positive for Olig2 and CC1 (Fig. 6N,O), and negative for Pax2 (Fig. 6P). Together, our lineage tracing analysis demonstrates that *Olig* gene-expressing progenitors give rise to PCs and DCN neurons, but rarely Pax2^+^ interneurons, in the cerebellum.

## Discussion

In this study, we aim to determine the neurogenic role of *Olig2* gene in the early developing cerebellum. We independently show that Olig2 is dynamically expressed in both the VZ and NTZ of the cerebellar primordium at early embryonic stages labeling different neuronal lineages during cerebellar neurogenesis. In particular, we show that deletion of *Olig2* alone, but not *Olig1* alone, specifically impairs PC generation of the cerebellum without substantially affecting other neuronal types. Furthermore, our long-term lineage tracing analysis indicates that *Olig* gene-expressing progenitors give rise to both DCN neurons and PCs, but rarely Pax2^+^ interneurons (Fig. 6), arguing against the “temporal identity transition” model of the cerebellar VZ progenitors that were recently proposed^15^.

### Potential mechanisms of Olig2 function in PC specification

The expression pattern relative to that of Pax2 has constrained Olig2 to the “pc2” domain of the cerebellar VZ where it could interact with other transcription factors^16^. *Ptf1a*-null mice exhibit a substantial loss of PCs and Pax2^+^ interneurons^9^, whereas *Mash1*-null mice do not show major defects on PC development^25^. Although *Ngn1* deletion has not been studied in the developing cerebellum in terms of its functional contribution to PC genesis^26^, *Ngn2* deletion results in a reduction of total number of PC as shown in a recent report^13^. In that report, Florio and colleagues conducted a detailed analysis of cell-cycle kinetics on cerebellar VZ progenitors and demonstrated that Ngn2 is mainly expressed in progenitors in the G1 phase getting ready to exit the cell cycle, and rapidly down-regulated in differentiated neurons^13^. We also showed that Olig2 is expressed in the late phase of the progenitor cell cycle (Fig. 5D) and also maintained even after cell cycle exit, since we observed 35.9% of Olig2^+^ cells overlapped with Lhx1/5^+^ postmitotic neurons in the E13.5 cerebellar VZ (Fig. 1E). Prolonged expression may allow Olig2 interact with different transcription factors including Ngn2 to function in PC generation.

Previous reports indicated that Olig2 and Ngn2 interact to regulate MN specification^27^,^28^,^29^. Olig2 function is believed to expand and prime pMN progenitors to MN fate whereas Ngn2 is to promote cell cycle exit and neuronal differentiation. Consistently, while *Ngn2*-null mice exhibit only impaired MN differentiation^30^, *Olig2* deletion abolishes MN phenotype^3^,^4^. The proliferative function of Olig2 is also demonstrated in the forebrain where a *Olig1/2* triplication mutant exhibits increased Ki67^+^ cells in the embryonic MGE, which can be rescued by a *Olig1/2*^+/−^ genotype^31^. Mechanistically, Olig2 and Ngn2 may interact at different levels. First, Olig2 protein can turn on *Ngn2* gene transcription^3^,^4^,^29^; second, Olig2 and Ngn2 can compete for binding to E-box DNA enhancer elements^27^. Therefore, these two factors may interact directly to coordinate neurogenesis. In a different scenario, Olig2 has been shown to suppress the transcription of *cell cycle dependent kinase inhibitor 1a* (*CDKN1A*) in neural stem cells, which in turn facilitates cell proliferation^32^. In addition, CDKs phosphorylates Ngn2 protein on multiple sites and inhibits its binding to E-box DNA fragments and thus transcriptional activity^33^. Therefore, these studies provide an alternative mechanistic pathway by which Olig2 and Ngn2 interact indirectly through cell cycle regulators to balance cell proliferation and neurogenesis. Olig2 and Ngn2 are both expressed and colocalized in the “pc2” domain of the cerebellar VZ^14^, allowing these similar mechanisms to occur during PC specification. In fact, *Olig2* deletion results in a slower rate of PC generation from the cerebellar VZ progenitors (Fig. 4) suggesting a defect of cell cycle kinetics, and *Ngn2*-null cerebellar VZ progenitors stall in the G1 phase of the cell cycle and thus produce fewer PCs^13^. This suggests that Olig2 and Ngn2 may functionally converge to cell cycle regulation of the cerebellar VZ progenitors. An interesting observation worthy of noting is that *Olig2* deletion induces only impaired PC generation in the cerebellum (this study) but a complete absence of MNs in the spinal cord^3^,^4^. This underlines differential contribution of Olig2 in the generation of PCs vs. MNs, and may be reflected by the distinct proliferative characteristic of Olig2-expressing progenitors in the cerebellar VZ (Fig. 5).

### Function of *Olig* genes in the generation of interneurons and PCs in the cerebellum

Seto *et al*. demonstrated clearly that *Olig1/2* double knockout (KO) drastically reduces (but not abolishes) PCs and induces an increase of Pax2^+^ interneurons generation in the developing cerebellum^15^. Furthermore, the authors did not observe any obvious phenotype in the *Olig2* single KO. However, our results indicate that the *Olig2* single KO mildly, but significantly, reduced PC generation with no detectable effect on cerebellar interneurons (Fig. 2). This raises an interesting possibility that the effect of *Olig* genes on PCs and interneurons generation is dose-dependent, i.e. PC generation is more sensitive to *Olig* gene function and prone to a decrease in *Olig2* single KO, whereas interneuron generation is less sensitive and requires deletion of both *Olig1* and *Olig2* to show an increase in number. We showed that *Olig* genes (at least *Olig2*) function in the late phase of the progenitor cell cycle (Fig. 5) and maintain a proper generation of PCs from these progenitors without affecting their overall proliferation (Fig. 4). However, the inhibitory effect of *Olig* genes in interneurons generation is unclear, but unlikely through Gsx1, since no change of *Gsx1* expression was observed in either *Olig* gene KO (Fig. 2) or overexpression (Fig. 7 in Seto *et al*.^15^). Further studies are required to delineate the underlying mechanisms.

### Olig2, a mainly neurogenic factor in the cerebellar VZ?

Given the strong expression of Olig2 in the VZ of the early developing cerebellum, we asked if the Olig2^+^ VZ progenitors generate oligodendrocytes after they give rise to GABAergic neuronal cell types. However, we were unable to detect PDGFRα^+^ or NG2^+^ OPCs by immunostaining between E11.5 and E14.5 in the cerebellum (data not shown). Furthermore, the cerebellar OPCs gradually appear starting from E14.5 when Olig2 expression already disappears in the VZ (Fig. S1). This expression correlation suggests that oligodendrocytes may not be generated in the cerebellar VZ locally. In fact, cerebellar oligodendrocytes are thought to originate from extracerebellar sources^25^,^34^,^35^. By electroporating a GFP construct into the cerebellar VZ of the mouse embryos, Grimaldi *et al*. demonstrated that oligodendrocytes are not derived from cerebellar VZ progenitors at E14.5. In the same study, E12.5 solid cerebellar tissues were transplanted into the P2 GFP mouse cerebellum and examined 12 days after. The authors found that the majority of Olig2^+^ OPCs in the graft are also GFP^+^, indicating that they are not generated locally, but from the donor^25^. Extracerebellar origin of oligodendrocytes is also suggested in a quail/chick chimeric study^35^. Therefore, combining these previous reports and our results, we propose that, unlike in the spinal cord where it specifies both MNs and oligodendrocytes, Olig2 expressed in the VZ may be a mainly neurogenic factor during early cerebellar development, and that the classic Olig2-mediated neuron-oligodendrocyte linkage may be uncoupled in the developing cerebellum.

### Long-term lineage tracing analysis does not support the “transition” model of cerebellar VZ progenitors

Based on a short-term lineage tracing and gene functional analyses, a “transition” model has been proposed to describe the relationship between cerebellar VZ progenitors of PCs and Pax2^+^ interneurons^15^. This model claims that during development, Olig2^+^ PC progenitors (PCPs) transition into Gsx1^+^ interneuron progenitors (PIPs) to generate Pax2^+^ interneurons in the cerebellar VZ, and that this timely transition is important to generate proper number of two cerebellar neuronal subtypes and is regulated positively by Gsx1 and negatively by Olig2 (Fig. 7 in Seto *et al*.^15^). According to this model, one would predict that lineally most, if not all, Pax2^+^ interneurons are derived from *Olig* gene-expressing PCPs. Unfortunately, our long-term genetic lineage tracing analysis does not support this model by showing that Pax2^+^ interneurons are rarely labeled in an either *Olig1-Cre* or *Olig2-Cre* reporter line indicating that Pax2^+^ interneurons may not be lineally related to Olig2^+^ PCPs. Furthermore, we, as well as Seto *et al*. themselves^14^, provide evidence that Olig2 is expressed in the late phase of cell cycle in progenitors that are ready to leave the cell cycle and differentiate into PCs. Therefore, we propose that Olig2^+^ cells are PC-committed progenitors and cannot transition to PIPs during cerebellar development. It is still possible that a transient expression of Olig2 during the presumptive PCP to PIP transition does not permit a threshold of cellular Cre accumulation to allow efficient Cre-mediated recombination and therefore negative reporter expression in the PIP of the *Olig2-Cre* reporter mice (Fig. 6). However, since most Olig2^+^ progenitors are late-phase progenitors and ready to leave the cell cycle, it is unlikely for them to re-enter the cell cycle and transition to PIP. Whether other VZ progenitors such as Olig2^−^/BrdU^+^ cells (probably Ptf1a^+^) are able to transition to PIPs is still an open question, but no strong evidence exists to support it yet. Alternatively, the observed cerebellar VZ progenitor phenotype^15^ can be explained by expansion and/or migration of the Gsx1^+^ PIPs while PCPs are exhausted by PC generation between E11.5 and E13.5. Our data cannot exclude a small-scale transition between different types of progenitors since we do observe a small number of Parvalbumin^+^ small interneurons (in the ML) that are labeled by *Olig2-Cre* (Fig. 6L), although this partial labeling of Pax2^+^ interneurons can also be explained by a partial overlapping expression pattern of the markers rather than a transition. Further investigations are needed to clarify the underlying mechanism.

## Conclusion

Our work demonstrates that Olig2 is expressed in the late phase of the VZ progenitor cell cycle in the developing cerebellum and functionally required for normal PC generation from cerebellar VZ progenitors. The “pro-neuronal” characteristic of Olig2 in the cerebellar VZ progenitors distinguishes its role from other CNS regions such as spinal cord where Olig2 regulates specification of both neurons and oligodendrocytes. In addition, our observations from *Cre-lox*-mediated long-term genetic lineage tracing analyses challenge the “cerebellar progenitor identity transition” model that has been recently proposed. In fact, our data indicate that the majority of Olig2^+^ cells in the cerebellar VZ are either PC-committed progenitors or newborn PCs, and they unlikely switch their progenitor identity to give rise to cerebellar interneurons, at least, not in a large scale.

## Methods

### Mouse lines and genotyping

The *Olig1-Cre*^23^ and *Olig2-Cre*^36^ mice were crossed with the *Z/EG21*^37^*, ROSA26;tdTomato* (Ai14)^38^*, or ROSA26;LacZ*^39^ reporter mice, to examine promoter-specific Cre activity. The *Olig1*^23^ and *Olig2*^3^ single gene knockout mice were obtained by crossing heterozygous mice. Genotyping of the mice were performed as previously described^3^,^23^,^40^. The Institutional Animal Care and Use Committee of West China 2^nd^ University Hospital at Sichuan University approved the use of the animals and the experiments. All procedures were carried out in accordance with the approved guidelines.

### RNA extraction and quantitative reverse transcriptase PCR (qRT-PCR)

Total RNA was isolated from micro-dissected E18.5 cerebellar tissue samples using TRIzol^®^ reagent, according to the manufacturer’s instructions (Invitrogen). qRT-PCR analysis of gene expression was performed as previously described^40^. The relative gene expression levels were normalized to that of the housekeeping gene *GAPDH*. The primer sequences of the target genes are shown in Table 1.

### *In vivo* BrdU pulse-labeling

Pulse-labeling of dividing cerebellar progenitors was carried out by intraperitoneal injection of BrdU (5-bromo-2′-deoxyuridine, Sigma) into timed pregnant mice at 100 mg/kg body weight. Mice were sacrificed 0.5, 2, 8 or 14  hours after injection. BrdU staining was performed by incubating frozen sections at 37 °C in 2 M hydrochloric acid (HCl) solution for 30 minutes before standard immunohistochemistry.

### Acute cerebellar cultures

The acute cerebellar cultures were prepared as described^40^. Briefly, embryonic cerebella were micro-dissected with meningeal membranes removed. Tissues were dissociated and plated at a density of 100,000 cells/coverslip. Cells were then fixed after 2 hours incubation at 37 °C with 5% CO_2_ and subjected to immunostaining.

### Immunohistochemistry, immunocytochemistry and fluorescence microscopy

Cerebellar frozen and vibratome sections were prepared as previously described^40^,^41^,^42^. Tissue sections or fixed cell cultures were incubated with monoclonal antibodies against BrdU (rat IgG, 1:200, Accurate), Olig2 (mouse IgG, 1:200, Millipore), APC (mouse IgG, clone CC-1, 1:200, Calbiochem) and NeuN (mouse IgG, 1:400, Millipore); polyclonal antibodies against β-Galactosidase (β-gal) (chicken IgY, 1:2000, Abcam), BLBP (rabbit IgG, 1:500, Abcam), Tbr1 (rabbit IgG, 1:400, Proteintech), Pax2 (rabbit IgG, 1:50, Proteintech), Pax6 (rabbit IgG, 1:100, Proteintech), GABA (rabbit IgG, 1:2000, Sigma), Parvalbumin (rabbit IgG, 1:800, ImmunoStar), Olig2 (rabbit IgG, 1:500, Millipore or guinea pig IgG, 1:500, a gift from Dr. Ben Novitch at UCLA), GFAP (chicken IgY, 1:1000, Aves or rabbit IgG, 1:2000, DAKO), Calbindin (rabbit IgG, 1:500, ImmunoStar), Calretinin (rabbit IgG, 1:500, ImmunoStar), cleaved caspase-3 (rabbit IgG, 1:1000, Cell Signaling), and DCX (guinea pig IgG, 1:1000, Millipore), followed by appropriate species-specific secondary antibodies (Molecular Probes). DAPI (10 μg/ml, Sigma) was included in the secondary antibody incubations to label nuclei. The tissue sections or cultured cells were then mounted in mounting medium (Zhong Shan-Golden Bridge Biotech, P.R. China) and analyzed by conventional (Nikon Eclipse T*i*) or confocal (Zeiss LSM 5 duo) fluorescence microscopy.

### Measurements and statistical analysis

For cell culture staining, in Fig. 2B,C, numbers of Calbindin^+^ cells were counted in multiple image fields of cultures with a similar cell density from at least 3 different animals for both controls and mutants; in Fig. 2D,E, percentage of Pax6^+^ cells were counted and calculated from 100 to 500 DAPI^+^ cells in multiple image fields of cultures from at least 3 animals for both controls and mutants. For section staining, in Fig. 2A, the number of Calbindin^+^ cells per cerebellar section were counted from 3 animals for both controls and mutants; in Fig. 3, the number of Pax2^+^ cells per cerebellar section were counted from 3 animals for both controls and mutants; in Fig. 4B, percentage of BrdU^+^ cells were calculated from more than 600 DAPI^+^ cells on multiple sections of at least 3 animals for both controls and mutants; in Fig. 4D, a total of more than 600 BrdU^+^ cells were counted, and percentage of Lhx1/5^+^ in BrdU^+^ cells were calculated; in Fig. 5C,D, percentage of marker-positive cells were calculated from more than 600 total cells on multiple sections of at least 3 animals. All data were presented as means ± standard deviation except for Fig. 5C,D where only means were presented. Statistical analysis was performed in Microsoft Excel using Student’s *t*-test.

## Additional Information

**How to cite this article**: Ju, J. *et al*. Olig2 regulates Purkinje cell generation in the early developing mouse cerebellum. *Sci. Rep*. **6**, 30711; doi: 10.1038/srep30711 (2016).

## Supplementary Material

Supplementary Information

## Figures and Tables

**Table 1:** Primers used for qRT-PCR.

Gene Name	Forward	Reverse
*Olig2*	*cgcaagctctccaagatcg*	*ctcaccagtcgcttcatctc*
*Olig1*	*tcctcatcctcatcctcttcc*	*tgttcctctttggcgtcg*
*Calbindin*	*attctgaaacgatctcccta*	*tctggctaccttccctta*
*RORα*	*cttcccctactgttccttcac*	*aaggtctgccacgttatctg*
*Pax6*	*ccctcaccaacacgtacag*	*tcataactccgcccattcac*
*Pax2*	*gatcctactccatcaacggg*	*gaccagatgtaaacctccacc*
*BLBP*	*acccgagttcctccagtt*	*tgccaccttcctgactga*
*Tbr1*	*cactcatcctctcctttctcttg*	*ggtgcaggttctagagtcaac*
*Brn2*	*caagtaggtaaggtccgaagc*	*tggtaaggttcagtggtaatgtc*
*Gsx1*	*ggtcctttatgcaacacgtcta*	*gcaagacctgatccctgttt*
