# Supplementary material for: Olig2 regulates Purkinje cell generation in the early developing mouse cerebellum
Source: Sci Rep. 2016 Jul 29;6:30711. doi: 10.1038/srep30711 (PMC4965836; doi:10.1038/srep30711)
Supplement: Supplementary Information [file srep30711-s1.pdf]

Supplemental material

Title: Olig2 regulates Purkinje cell generation in the early developing mouse cerebellum

Jun Ju[1,4,†], Qian Liu[1,†], Yang Zhang[1,4], Yuanxiu Liu[1,4], Mei Jiang[1], Liguo Zhang[5], Xuelian He[5], Chenchen Peng[1,4], Tao Zheng[1], Q. Richard Lu[5] and Hedong Li[1,2,3,*]

[1]West China Developmental & Stem Cell Institute, [2]Department of Obstetric & Gynecologic and Pediatric, [3]Key Laboratory of Obstetric & Gynecologic and Pediatric Diseases and Birth Defects, Ministry of Education, West China Second University Hospital, [4]School of Life Science, Sichuan University, Chengdu 610041, P.R. China, [5]Department of Pediatrics, Cincinnati Children's Hospital Medical Center, Cincinnati, Ohio 45229, USA

[†]These authors contributed equally to this work.

Supplemental Figure 1:

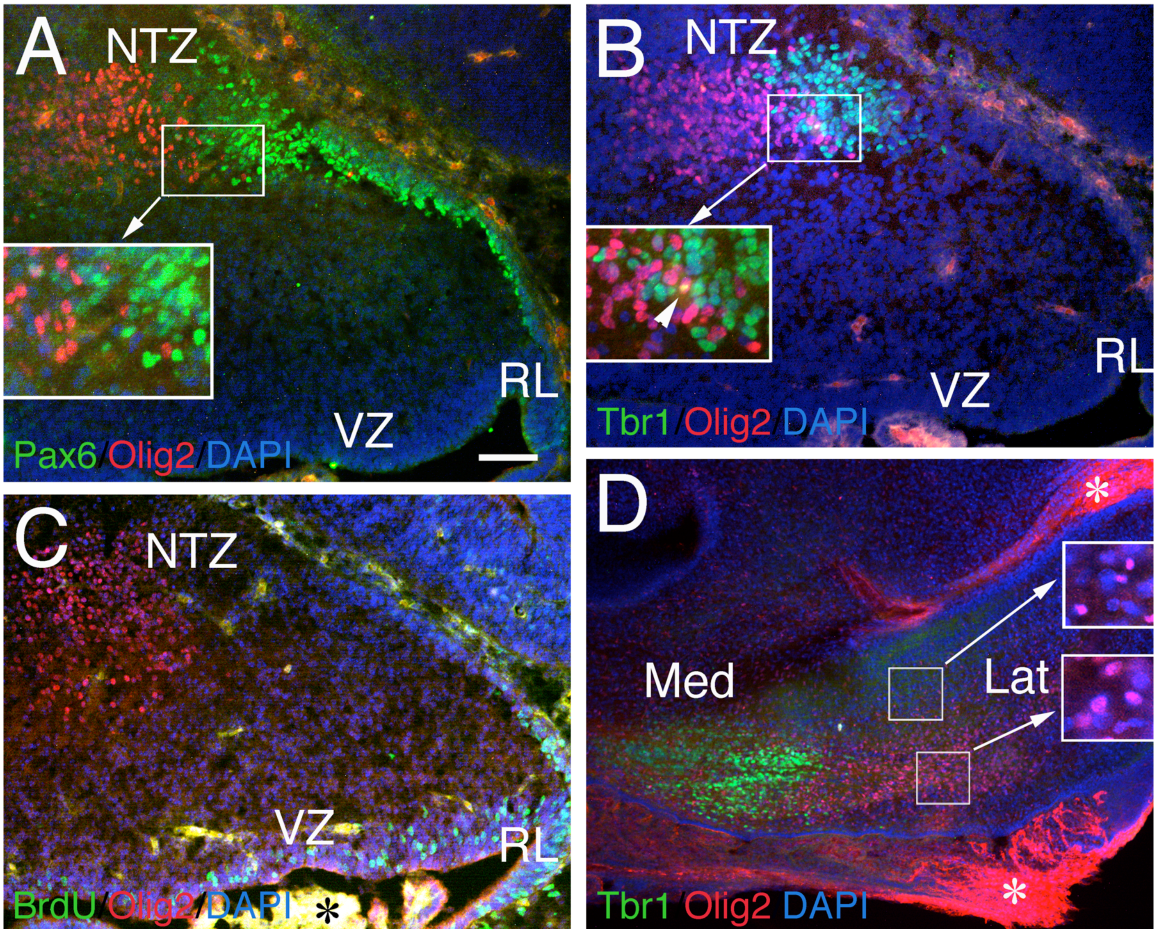

Figure S1. **The NTZ Olig2$^+$ cells represent a distinct subpopulation of DCN neurons in the embryonic cerebellum.** Mid-sagittal sections of the E14.5 (**A-C**) and coronal sections of the E18.5 (**D**) wild-type cerebella are immunostained. The NTZ Olig2 and Pax6 (to label GNPs) show a non-overlapping pattern (**A**). Between the Olig2$^+$ and Pax6$^+$ domains, there are a group of cells that are negative for both markers, but positive for Tbr1 (to label a subset of DCN neurons) (**B**). Higher magnification views of the boxed regions are shown as inserts in (**A** and **B**). Although the Olig2$^+$ and Tbr1$^+$ domains are closer in position with double positive cells being occasionally seen (**B**, insert, arrowhead), they are largely non-overlapping. In addition, the NTZ Olig2$^+$ cells are nearly all postmitotic as indicated by their inability to take up BrdU in a BrdU-pulse labeling experiment (**C**). Notably, a number of BrdU$^+$ cells can still be found in the VZ, and yet Olig2 expression has already disappeared in this region at this developmental stage. At E18.5, while Tbr1$^+$ neurons concentrate to the medial (Med) part of the DCN, Olig2 expression is more towards the lateral (Lat) region (**D**). Two morphologically distinct Olig2$^+$ cells are found with smaller cells being located in the periphery of the Olig2$^+$ domain and bigger cells being in the core (**D**, inserts). Non-specific staining signals from the choroids plexus and a representative blood vessel are indicated by asterisks in (**C**) and (**D**). VZ, ventricular zone; RL, rhombic lip; NTZ, nuclear transitory zone. Scale bar: 80 μm in **A-C**; 160 μm in **D**.
